# Racial/ethnic and sexual behavior disparities in rates of sexually transmitted infections, San Francisco, 1999-2008

**DOI:** 10.1186/1471-2458-10-315

**Published:** 2010-06-06

**Authors:** Hyman M Scott, Kyle T Bernstein, Henry F Raymond, Robert Kohn, Jeffrey D Klausner

**Affiliations:** 1 Department of Medicine, University of California, San Francisco, California, USA; 2 STD Prevention and Control Services, San Francisco Department of Public Health, 1360 Mission St., Suite 401, San Francisco, California, USA; 3 HIV Epidemiology Section, AIDS Office, San Francisco Department of Public Health, 25 Van Ness, Suite 500, San Francisco, California, USA

## Abstract

**Background:**

Racial/ethnic minorities and men who have sex with men (MSM) represent populations with disparate sexually transmitted infection (STI) rates. While race-specific STI rates have been widely reported, STI rates among MSM is often challenging given the absence of MSM population estimates. We evaluated the race-specific rates of chlamydia and gonorrhea among MSM and non-MSM in San Francisco between 1999-2008.

**Methods:**

2000 US Census data for San Francisco was used to estimate the number of African-American, Asian/Pacific Islander, Hispanic, and white males. Data from National HIV Behavioral Surveillance (NHBS) MSM 1, conducted in 2004, was used to estimate the total number of MSM in San Francisco and the size of race/ethnic sub-populations of MSM. Non-MSM estimates were calculated by subtracting the number of estimated MSM from the total number of males residing in San Francisco. Rates of MSM and non-MSM gonorrhea and chlamydia reported between 1999 and 2008 were stratified by race/ethnicity. Ratios of MSM and non-MSM rates of morbidity were calculated by race/ethnicity.

**Results:**

Between 1999-2008, MSM accounted for 72% of gonorrhea cases and 51% of chlamydia cases. Throughout the study period, African-American MSM had the highest chlamydia rate with 606 cases per 100,000 in 1999 increasing to 2067 cases per 100,000 in 2008. Asian/Pacific Islander MSM consistently had the lowest rate among MSM with1003 cases per 100,000 in 2008. The ratio of MSM/non-MSM for chlamydia was highest among whites 11.6 (95% CI: 8.8-14.4) and Asian/Pacific Islanders 8.6 (95% CI: 6.2-11), and lowest among African-Americans 1.53 (95% CI: 1.2-1.9) and Hispanics 4.43 (95% CI: 2.8-6.0). Gonorrhea rates were similar for African-American, white, and Hispanic MSM between 2137-2441 cases per 100,000 in 2008. Asian/Pacific Islander MSM had the lowest gonorrhea rate with 865 cases per 100,000 in 2008. The ratio of MSM/non-MSM for gonorrhea was highest among whites 11.6 (95% CI: 8.8-14.4) and Asian/Pacific Islanders 8.6 (95% CI: 6.2-11), and lowest among African-Americans 1.53 (95% CI: 1.2-1.9) and Hispanics 4.43 (95% CI: 2.8-6.0).

**Conclusions:**

For all racial/ethnic groups in San Francisco, MSM carried a substantially higher burden of STIs compared to non-MSM except among African-American men. These racial and sexual behavior disparities warrant further public health attention and resources.

## Background

Health disparities among racial and ethnic minorities have been well documented in the literature, particularly among Sexually Transmitted Infections (STIs) [1]. African-Americans have gonorrhea and chlamydia rates that are eighteen and eight times higher than whites respectively [2]. Similar disparities have been documented in San Francisco where African-Americans have significantly higher rates of gonorrhea and chlamydia compared to their white counterparts [3]. Men who have sex with men (MSM), also represent a population with disparate rates of STI/HIV compared to heterosexual men [4]. Several studies have demonstrated the increased incidence of STIs among African-American MSM in relation to HIV risk [5-10]. Furthermore, an overall lower incidence of STIs among Asian/Pacific Islanders has been reported [11]. The US Census does not currently capture data on sexual orientation or identity; as a result other methodologies are required to enumerate these populations [12]. Comparing population based STI trends among MSM is challenging given populations estimates of MSM overall, or stratified by race, are often unavailable. Consequently most studies utilize research samples, not county-wide morbidity data, and no study has looked at a comparison of men from all major racial/ethnic groups based on sexual behavior.

Racial/ethnic and sexual orientation specific prevalence of STIs are essential in determining the excess burden that some racial groups experience but also the increased STI burden that MSM have over other men. These data have broad implications including provision and targeting of prevention and treatment programs to address health and healthcare disparities.

The objectives of this study were to evaluate the race-specific rates of chlamydia and gonorrhea among men in San Francisco.

## Methods

As per Title 17, California Code of Regulations §2500, §2593, §2641-2643, §2800-2812, lab and provider reporting of chlamydia and gonorrhea to the San Francisco Department of Public Health is mandated. Data on the case's race/ethnicity (Hispanic/Latino, African American/Black, Asian/Pacific Islander (including Asian-Indian, Cambodian, Chinese, Filipino, Guamanian, Hawaiian, Japanese, Korean, Loatian, Samoan, Vietnamese, or "Other"), Native American/Alaskan Native, White, or "Other") and genders of sex partners are collected through routine provider and lab based reporting. In San Francisco, all STI morbidity among males is electronically matched to any prior episode of the patient (STI clinic visit, prior reported STI morbidity) to determine if the patient has documentation of reporting a male partner, a sexual identity of gay, or a diagnosis of rectal gonorrhea or rectal chlamydia. These data date back to the 1970s and we assumed that sexual orientation was fixed; i.e., that any previous report of having a male sex partner, gay sexual identity, or rectal STI was carried forward in time to any subsequent morbidity. Men who reported both male and female partners were included in our definition of MSM. All male reports for gonorrhea or chlamydia with no history of a male partner, gay identity, or a rectal infection were assumed to be non-MSM. All male gonorrhea and chlamydia morbidity at any site (urogenital, rectal or pharyngeal) among San Francisco residents that was separated by more than 30 days reported between 1999 and 2008 were included in the analysis.

Data from National HIV Behavioral Surveillance (NHBS) MSM 1, conducted in 2004, was used to estimate the total number of MSM in San Francisco [13]. Subsequently, subpopulation sizes were projected using the total population size estimate multiplied by the proportion of sub-populations sampled in NHBS MSM1. For example, based on NHBS MSM1 data there are an estimated 57,218 MSM in San Francisco. Fifty-six percent of these MSM were white, providing a population of estimate of 31,788 white MSM. 2000 US Census data for San Francisco was used to estimate the number of African-American, Asian/Pacific Islander, Hispanic, and white males residing in San Francisco [12]. The race/ethnicity specific non-MSM estimates were calculated by subtracting the number of estimated MSM from the total number of males residing in San Francisco.

Race/ethnicity-specific rates of MSM and non-MSM gonorrhea and chlamydia were estimated by dividing the number of reported cases in each MSM and racial/ethnic group by the estimated San Francisco population for each group. MSM to non-MSM racial/ethnic specific rate ratios were also estimated. As these were de-identified, publically available surveillance data used for public health purposes, this study was considered exempt from human-subjects considerations in accordance with the Code of Federal Regulations, Title 45, and by the Committee on Human Research at University of California, San Francisco.

## Results

In total, between 1999 and 2008, there were 15,418 case of gonorrhea and 14,197 cases of chlamydia reported in San Francisco. MSM accounted for 72% of the gonorrhea cases and 51% of the chlamydia cases during that period. In San Francisco, the estimated number of white MSM was 31,788, African-American MSM 4,450, Hispanic MSM 10,808, and Asian/Pacific Islander MSM 10,127. Among non-MSM in San Francisco, an estimated 147,538 were white, 25,511 were African-American, 47,047 are Hispanic, and 104,810 were Asian/Pacific Islander.

### Chlamydia

**Figure 1:**
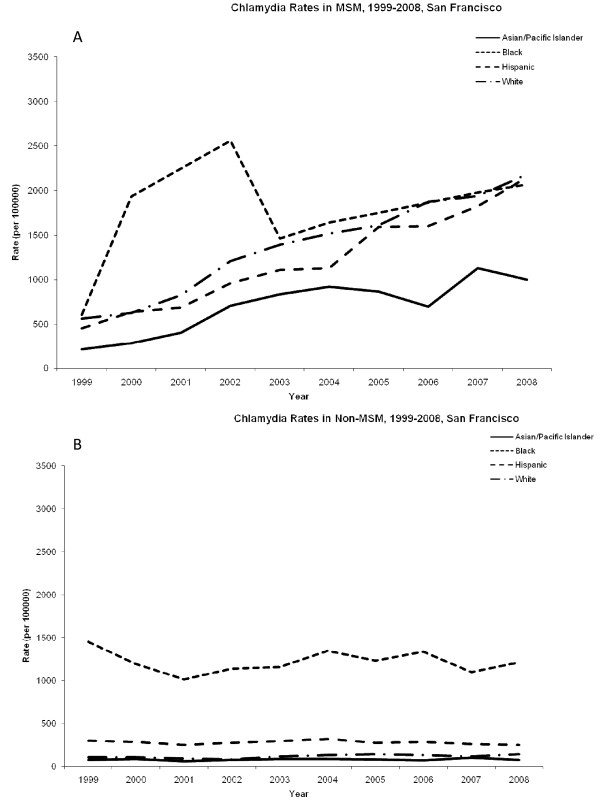
**Rates of chlamydia among men in San Francisco, 1999-2008**. (A) Cases of chlamydia among men who have sex with men (MSM) per 100,000. (B) Cases of chlamydia among non-MSM per 100,000.

**Figure 2:**
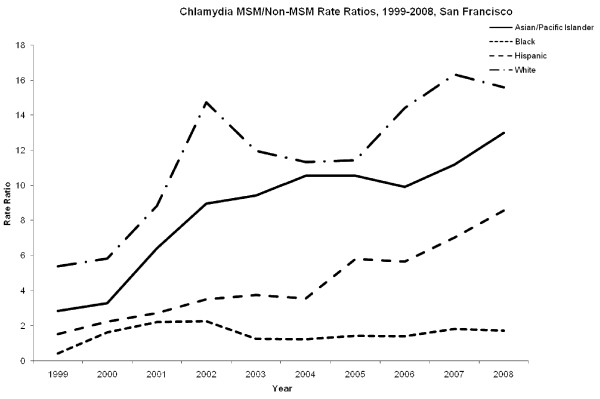
**MSM/non-MSM ratio of chlamydia in San Francsico, 1999-2008**. MSM to non-MSM racial/ethnic specific rate ratios for chlamydia.

In San Francisco, case rates of chlamydia among MSM increased for all racial groups during the study period (Figure 1A). African-American MSM consistently had the highest rates of chlamydia with 606 cases per 100,000 in 1999 and 2067 cases per 100,000 in 2008. Asian/Pacific Islanders had the lowest case rate during the study period with 1003 cases per 100,000 in 2008. While rates of MSM chlamydia appeared to increase over the analytic period, non-MSM chlamydia rates remained largely stable (Figure 1B). African-American non-MSM also had the highest rates of chlamydia with 1450 cases per 100,000 in 1999 and 1211 cases per 100,000 in 2008. Non-MSM in all other racial groups had at least a four fold lower case rate of chlamydia compared to African-Americans. The rate ratio of MSM/non-MSM was highest among Whites 11.6 (95% CI: 8.8-14.4) and Asian/Pacific Islanders 8.6 (95% CI: 6.2-11) and lowest among African-Americans 1.53 (95% CI: 1.2-1.9) and Hispanics 4.43 (95% CI: 2.8-6.0) (Figure 2).

Between 1999 and 2003, 65% to 81% of chlamydia cases in MSM were diagnosed at the San Francisco STI Clinic (City Clinic). However, in 2007 only 55% of cases were diagnosed at City Clinic and 22% were diagnosed by primary care physicians (compared to 7% in 1999). In contrast, non-MSM were diagnosed in similar proportions at various clinic settings including City Clinic, primary care offices, hospital, and outreach settings throughout the study period.

### Gonorrhea

**Figure 3:**
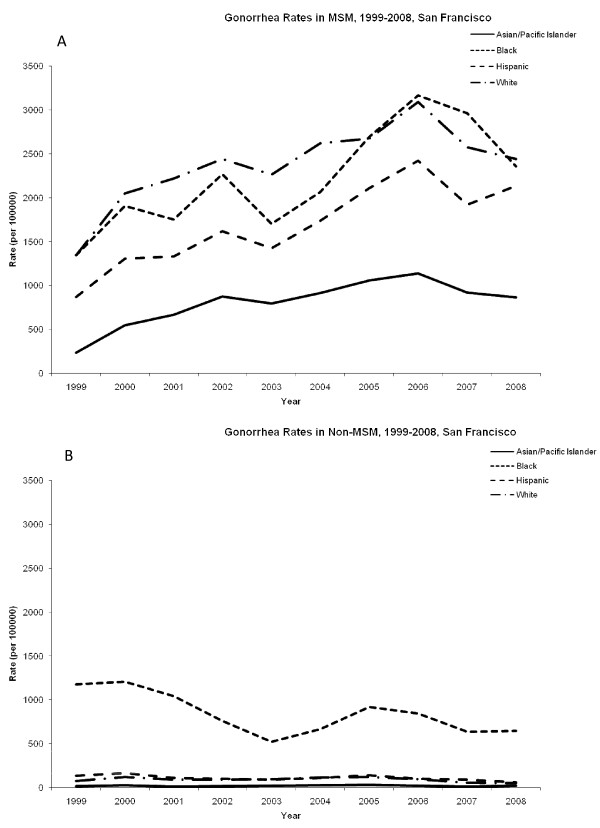
**Rates of gonorrhea among men in San Francisco, 1999-2008**. (A) Cases of gonorrhea among men who have sex with men (MSM) per 100,000. (B) Cases of chlamydia among non-MSM per 100,000.

**Figure 4:**
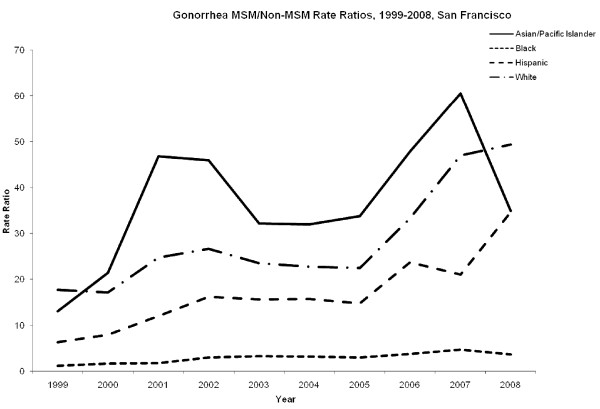
**MSM/non-MSM ratio of gonorrhea in San Francisco, 1999-2008**. MSM to non-MSM racial/ethnic specific rate ratios for gonorrhea.

Rates of gonorrhea among MSM increased between 1999 and 2008 (Figure 3A). In 2008, African-Americans and Whites had similar rates of gonorrhea at 2360 per 100,000 and 2441 cases per 100,000 respectively. Hispanics had similarly high rates of gonorrhea (2137 cases per 100,000) and Asian/Pacific Islanders had the lowest gonorrhea rates (865 cases per 100,000) in 2008. Among non-MSM, African-Americans had case rates of gonorrhea at least five fold higher than all other racial groups during the study period (Figure 3B). The rate ratio of MSM/non-MSM was highest among Asian/Pacific Islanders 36.8 (95% CI: 26.9-46.7) and Whites 28.5 (95% CI: 20.4-36.57), and lowest among African-Americans 2.9 (95% CI: 2.1-3.7) and Hispanics 16.8 (95% CI: 10.9-22.7) (Figure 4).

Like chlamydia, approximately 70% of gonorrhea cases were diagnosed in City Clinic between 1999 and 2003 and 5% to 10% of cases diagnosed in primary care offices. By 2007, 58% of cases were diagnosed in City Clinic and 24% were diagnosed in primary care offices. Among non-MSM, approximately two thirds of gonorrhea cases were diagnosed at City Clinic and hospital settings between 1999 and 2003. However, by 2007, 38% of cases were diagnosed in primary care offices and 15% diagnosed at either City Clinic or hospital settings.

## Discussion

In measurement of disease burden, rates are superior to case counts as they account for the underlying population at risk and provide a measure of disease intensity in a sub-population. Often MSM specific STI rates are difficult to calculate given that population estimates of MSM are often unavailable. Here we report on MSM and non-MSM racial/ethnic specific rates of STIs in San Francisco. Between 1999 and 2008, rates of MSM chlamydia and gonorrhea increased, while non-MSM rates of these STIs remained largely stable. These increases in STIs are consistent with previous reports in San Francisco and other cities [11,14]. African-American, Hispanic, and White MSM had similar rates of chlamydia and gonorrhea while Asian/Pacific Islander MSM had the lowest rates during the study period. The MSM/non-MSM ratio was lowest for African-American men and highest for White and Asian/Pacific Islander men. While African-American MSM had comparable rates of chlamydia and gonorrhea to other racial/ethnic groups, African American non-MSM had rates up to five times higher than these groups.

Reducing racial disparities is a national public health priority. STIs, specifically gonorrhea, represent some of the largest racial/ethnic disparities among reportable conditions [4]. Our data highlight not only the disparities that exist across race and ethnicity, but also among subpopulations of MSM and non-MSM. These data identify important trends in chlamydia and gonorrhea morbidity that are often masked when all male morbidity is examined without regard to sexual behavior.

Furthermore, the data presented here suggest that the disparities paradigm be expanded to include sexual behavior. For all racial/ethnic groups, MSM had significantly higher rates of chlamydia and gonorrhea compared to non-MSM. In San Francisco, MSM are significantly burdened with STIs and these disparities warrant public health attention and resources.

San Francisco STD Prevention and Control Services recommends that all sexually active MSM be screened for syphilis, chlamydia, and gonorrhea every three to six months. In San Francisco, routine screening for gonorrhea and chlamydia at the urogenital, pharyngeal, and rectal sites is widely available for MSM and has been shown to lead to significantly higher rates of gonorrhea and chlamydia diagnoses, primarily through diagnosis of asymptomatic infections [15,16]. After increased rates of syphilis were reported in 2001 and 2002, there have been multiple public health campaigns for routine STI screening of MSM which may partially explain the increased percentage of cases diagnosed in primary care offices during our study period. Additionally, San Francisco STD Prevention and Control Services has worked over the prior decade to ensure that STD screening is incorporated into routine HIV care for MSM in San Francisco.

Based on lower sexual risk behavior such screening is not routinely indicated in non-MSM and the higher rate of STIs among African-American non-MSM in San Francisco, while previously documented, is not well understood. Although several studies have shown that African-American MSM are less likely compared to other racial groups to self-identify as "gay" or "bisexual" we used data on gender of sex partners and not sexual identity to define MSM [17-19]. In addition, we found the percentage of African-American men classified as MSM was similar to that of white and Hispanic men, which would not support misclassification of sexual behavior as a sufficient explanation. Sexual networks, social context, and other contextual factors have been identified as important factors in racial STI disparities in other regions of the US [20-23]. It is unclear how these more macro level factors may function differently (if at all) between MSM and non-MSM.

Our analysis has several limitations worth noting. Our STI case rates and MSM/non-MSM ratios are dependent on estimation of population sizes for both MSM and non-MSM in San Francisco. Using the National NHBS and 2000 census data, we calculated a midpoint estimate and assume that the size of each population is relatively stable over the time period of analysis (1999-2008). Comparison of the 2000 census data to the 2006-2008 American Community Survey data for San Francisco County did not reveal any significant demographic shifts [24]. Furthermore, the NHBS sample size of MSM of color was too small to make meaningful comparisons between groups of MSM [25-29]. In the beginning of our study period, up to 81% of chlamydia cases and 70% of gonorrhea cases were diagnosed in the public STI clinic. There are data that STI rates among MSM attending public STI clinics are higher than other clinical sites which could lead to an over estimation of STI rates [30,31]. However, we believe that the gonorrhea and chlamydia surveillance systems in San Francisco are robust, as electronic laboratory reporting has been integrated into routine surveillance activities. Additionally, our definition of MSM was considered fixed. If a male patient reported male partners in 1990 during an STD clinic visit or public health disease control interview, or on a morbidity report, all subsequent morbidity was classified as MSM. As a result, men who have not had recent male exposures would be misclassified as MSM. Finally, San Francisco is a unique urban environment and is residence to a large population of MSM. Therefore, these findings may have limited generalizibility. However, we believe the methods we employed could be used in other health jurisdictions to develop locally valuable measures of disease burden and disparity.

## Conclusions

We capitalized on our existing data infrastructure and locally available data to examine population-based estimates of the rates of gonorrhea and chlamydia among men in San Francisco. The rates of STIs among MSM continue to rise, while the rates among non-MSM have been stable. MSM carried a substantially higher burden of STIs compared to non-MSM except among African-American men. The lower MSM/non-MSM ratio among African-Americans was driven primarily by the high rates among African American non-MSM. This study was the first population based estimate of the burden of gonorrhea and chlamydia among men in San Francisco. Utilizing the MSM population estimates of San Francisco and sexual partner gender data acquired at the time of testing, we report racial disparities that mirror those previously documented in the literature. We also suggest that among African-American men the gender of sexual partners is less of a determinant of gonorrhea or chlamydia rates than for other racial groups. This phenomenon warrants further research to establish contributing factors in order to develop effective, appropriate prevention strategies.

## Competing interests

HS, KB, HR, RK, and JK declare that they have no competing interests.

## Authors' contributions

HS contributed to developing the idea for the study, assisted with data analysis, and developed the manuscript. KB provided advice and guidance for the study, assisted with data analysis, and contributed to the manuscript. HR developed MSM population estimates and contributed to the manuscript. RK assisted with data analysis. JK develop the idea for the study, oversaw all aspects of the study, and contributed to the manuscript. All authors read and approved the final manuscript.

## Pre-publication history

The pre-publication history for this paper can be accessed here:

http://www.biomedcentral.com/1471-2458/10/315/prepub
